# Hospital Expenditure.—The Commissariat

**Published:** 1896-06-27

**Authors:** 


### THE INSTITUTIONAL WORKSHOP.

### HOSPITAL EXPENDITURE.—THE COMMISSARIAT.

### XVII.—THE LONDON MEAT TRADE.

The variations in the price of meat in metropolitan poor-law infirmaries are fully as remarkable as those in metropolitan hospitals. It is not practicable to institute any comparison between the two sets of prices, because the latest return from the Local Government Board dates as far back as 1890. But the figures quoted have an important bearing on the question of the meat supply to large institutions. We again take the stock articles of hospital consumption, the price quoted being per stone of 14 lbs. :—

| UNION OR PARISH. | MUTTON. 1890. s. d. | MUTTON. 1886. s. d. | LEG OF BEEF. 1890. s. d. | LEG OF BEEF. 1886. s. d. |
|---|---|---|---|---|
| Camberwell | 8 11½ | 7 6 | — | — |
| Chelsea | 4 0 | 8 2 | — | — |
| | 3 5 | 7 7 | — | — |
| | | 7 0 | — | — |
| Fulham | 7 1½ | 7 6 | 7 0½ | 4 0 |
| St. George's-in-the-East | 7 1¾ | 7 5 | — | 6 6 |
| St. George's, Fulham Rd. | 7 5¼ | 8 10½ | 4 5 | — |
| Greenwich | 6 3½ | 7 2½ | 2 11 | 3 5½ |
| Hackney | 7 8½ | 7 2 | 6 5 | — |
| Hampstead | 6 6¾ | — | 2 11 | — |
| Holborn | 7 2 | 8 8 | — | — |
| Islington | 8 2 | 8 10 | 3 11 | 4 2½ |
| | | 6 4½ | — | — |
| Kensington | 9 2 | 9 9 | 4 10 | 3 8 |
| Lambeth | 8 0¼ | 7 6 | — | — |
| | 7 5¼ | — | — | — |
| London, City of | 5 10 | 6 2¼ | 8 2 | — |
| St. Marylebone | 7 10½ | 8 3¾ | 5 6½ | 3 6 |
| Mile End Old Town | 8 5½ | 8 4 | 2 11 | 3 6 |
| St. Olave's | 8 1 | 9 4 | 3 2½ | 3 6 |
| Paddington | 7 0½ | 7 1 | — | 4 1½ |
| St Pancras | 8 10¾ | 6 4 | — | — |
| St. Saviour's | 7 5¼ | 7 1 | 5 11¾ | 3 6 |
| Shoreditch | 8 8 | 8 8¾ | — | 3 6 |
| Wandsworth & Clapham | 5 8 | 7 6 | 6 0½ | — |
| Whitechapel | 8 [illegible]½ | 7 1¼ | — | — |
| Woolwich | 8 9 | 9 0½ | — | — |
| | 8 0¼ | — | — | — |
| | 5 10 | — | — | — |

These figures are commended to the attention of any would-be reformer who may cherish the sanguine anticipation that handing the hospitals over to the control of the ratepayer would instantly abolish all anomalies of expenditure. It can hardly be disputed that a variation of over 5s. a stone, as between Chelsea and Kensington, in the price of mutton is in the last degree unsatisfactory, considering that precisely the same class of persons is catered for, and that the supply is furnished to adjoining districts of the same town.

It is perfectly clear that a widely-varying standard exists both among managers of hospitals and Poor-law Guardians with regard to the quality of meat which should be provided for the consumption of the sick, and that in many instances an extraordinary ignorance subsists of the current prices of the meat market.

We are forced to the conviction that the purchase of meat in the large quantities required for institutions demands exceptional skill and training, and that to delegate the choice to contractors selected by those who are quite innocent of the intricacies of the market cannot fail to open the door to every form of abuse.

The remedy lies in the direction of co-operation. In no other way can the full responsibility of the hospital with regard to the meat supply be fulfilled. The establishment of a central store charged with the selection and slaughtering of the animal, and with the preparation and distribution of the meat, can alone relieve managers of the unsatisfactory dilemma in which they now stand, and would, if properly organised and supported, ensure the supply of meat of sound quality and establish a fair level of prices. We say a fair level, because not all managers understand that when prices are quoted below the ordinary market level the contractor is bound to get his profit in an underhand manner, by furnishing the meat wet, or in other ways well understood by the trade.

It is well known that in many large towns in France a hospital co-operative store has been found to answer well. In Brussels, where the hospital meat store is specially well organised, the amount consumed is not sufficient to warrant any comparison with London requirements : but in Paris the quantity of meat required for the Assistance Publique approximates very nearly to what is consumed in London voluntary hospitals, and no difficulty has been experienced in meeting the demand.

It cannot be too clearly emphasised that such a central hospital meat *depôt*, although it would undoubtedly tend in the direction of economy and the lowering (in most cases) of prices, would exercise its highest work in presenting a guarantee of the sound quality of all the meat distributed. It is almost incredible that hospitals as a whole have been content to cultivate an absolute indifference with reference to the health and condition of the animals from which the meat supply used in the treatment of disease is drawn, and the almost equally important circumstances under which the slaughtering and preparation of the meat for market are carried on. In the Paris store a certificate of the previous soundness and present good condition of each bullock at the time of consignment is required, and in view of the immense number of unsatisfactory, not to mention actually diseased, animals which are introduced into the market, this precaution must be considered essential. The abattoir again of the Assistance Publique is a model institution of its kind. In London it is not too much to expect that the hospitals shall take a foremost place in attempting to reform those abuses in slaughter-houses which are too horrible to bear open discussion. The fact is that the slaughter and preparation for the market of any considerable number of animals is a process which demands spacious and specially adapted premises, apparatus accurately adjusted and manipulated, and the most rigid enforcement of sanitary law if the risk of brutality towards the animal, of deterioration in the meat, and annoyance to the neighbourhood is to be reduced to a minimum. Looking to the great voluntary hospitals as leaders in the cause of humanity and hygienic progress, we cannot fail to be struck by their absolute indifference to a question which affects them so nearly, and to read the explanation of so conspicuous a neglect in that fatal want of unity and cohesion which has paved the way for countless anomalies in the history of medical charity.

It goes without saying that any such co-operative hospital store should comprise a department for the supply of foreign meat. The ignorance which prevails on the subject of the meat trade is nowhere more conspicuous than in this direction, and skilled selection is nowhere more urgently required. How many hospital managers, for instance, could distinguish between a leg of the best Canterbury mutton, fetching sevenpence or eightpence per pound, and a leg of Australian mutton from the merino sheep, which can be bought in the market at twopence per pound, and in many London shops at threepence? And how many could at a glance tell either of these from English Southdown at 10d. or 1s. per pound? And yet in the eyes of an expert buyer, to use his own words, "there is no more danger of confusing one for another than of mistaking a cabbage for a rose." Many persons not being experts fail to detect the difference when served at table, and the point is, not so much which shall the hospital buy—a question each must answer for himself—as how shall it ensure that, having paid for the one, it does not get the other.

What the hospitals want is access to a store supplied by trained buyers, acting not in the interests of the contractor but of the hospital; a store from which trade jobbery would be entirely excluded, and in which the trade subscriber should have no more influence than another, where each institution could procure the particular description of meat found to answer best the needs of the inmates, and be certain of getting exactly the stipulated quality at the lowest market prices.

